# A method of ‘speed coefficients’ for biochemical model reduction applied to the NF-$\upkappa$B system

**DOI:** 10.1007/s00285-014-0775-x

**Published:** 2014-03-22

**Authors:** Simon West, Lloyd J. Bridge, Michael R. H. White, Pawel Paszek, Vadim N. Biktashev

**Affiliations:** 1 Institute of Integrative Biology, University of Liverpool, Crown Street, L69 7ZB Liverpool, UK; 2 Department of Mathematics, Swansea University, Singleton Park, Swansea, SA2 8PP UK; 3 Faculty of Life Science, University of Manchester, Oxford Road, Manchester , M13 9PT UK; 4 College of Engineering, Mathematics and Physical Sciences, University of Exeter, North Park Road, Exeter, EX4 4QF UK

**Keywords:** Model reduction, Characteristic timescales, Signalling networks, Nuclear Factor kappa B, 92-08, 92C42

## Abstract

The relationship between components of biochemical network and the resulting dynamics of the overall system is a key focus of computational biology. However, as these networks and resulting mathematical models are inherently complex and non-linear, the understanding of this relationship becomes challenging. Among many approaches, model reduction methods provide an avenue to extract components responsible for the key dynamical features of the system. Unfortunately, these approaches often require intuition to apply. In this manuscript we propose a practical algorithm for the reduction of biochemical reaction systems using fast-slow asymptotics. This method allows the ranking of system variables according to how quickly they approach their momentary steady state, thus selecting the fastest for a steady state approximation. We applied this method to derive models of the Nuclear Factor kappa B network, a key regulator of the immune response that exhibits oscillatory dynamics. Analyses with respect to two specific solutions, which corresponded to different experimental conditions identified different components of the system that were responsible for the respective dynamics. This is an important demonstration of how reduction methods that provide approximations around a specific steady state, could be utilised in order to gain a better understanding of network topology in a broader context.

## Introduction

Biological systems are inherently complex. They are governed by a large number of functionally diverse components, which interact selectively and nonlinearly to achieve coherent outcomes (Kitano 2002). Systems biology addresses this complexity by integrating biological experiments with computational methods, to understand how the components of a system interact and contribute to the biological function. However, the dynamical models that represent biological systems can often have high-dimensional state space and depend on a large number of parameters. Understanding the relationships between structure, parameters and function of such large systems is often a challenging and computationally intensive task.

**Fig. 1:**
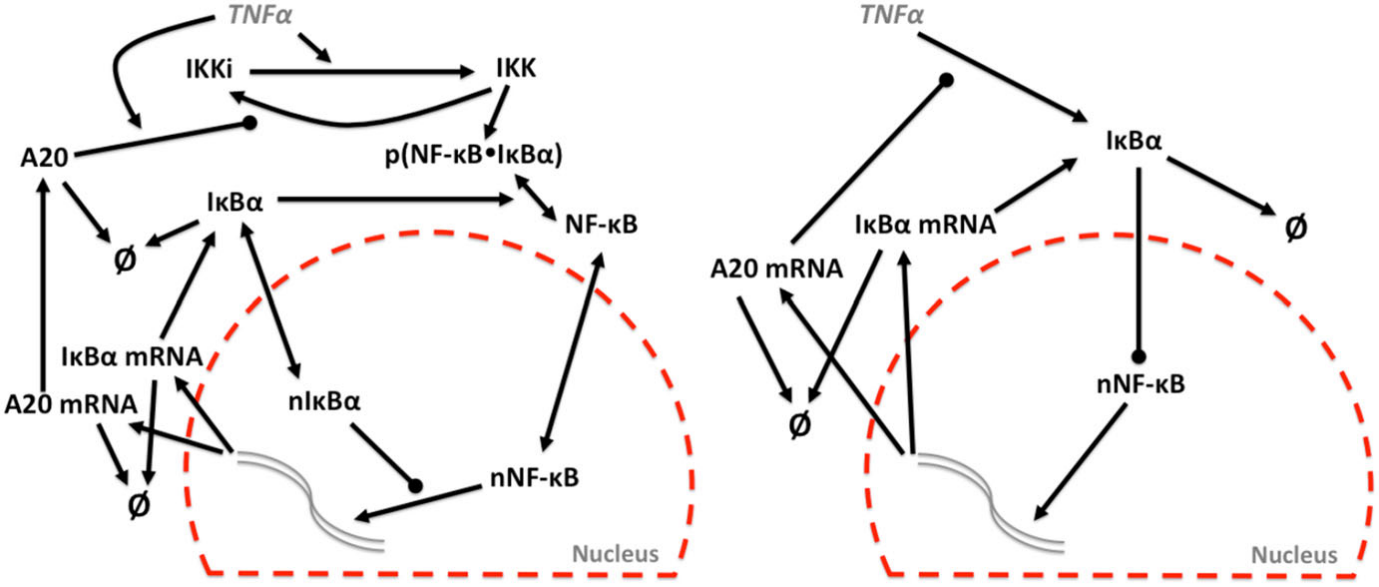
Network diagram of the Simplified Model (derived from Ashall et al. (2009)) and the minimal model of the NF-$\upkappa$Bsystem. Time-dependent variables present in each model are depicted with *black colour*. *Pointed* and *round arrowheads* represent activating and inhibitory reactions, respectively. In unstimulated conditions NF-$\upkappa$B is sequestered in the cytoplasm by association with I$\upkappa$B$\upalpha$ inhibitors. Stimulation with TNF$\upalpha$ (by changing $k_{24}=1$ from 0) causes activation of the IKK kinase, and subsequently degradation of I$\upkappa$B$\upalpha$ and translocation of free NF-$\upkappa$B to the nucleus. Nuclear NF-$\upkappa$B induces transcription of I$\upkappa$B$\upalpha$ and A20. Once synthesised I$\upkappa$B$\upalpha$ is able to bind to NF-$\upkappa$B and return it to the cytoplasm, while A20 inhibits the IKK activity

One example of such a complex and high-dimensional system is the signalling network of the nuclear factor kappa B (NF-$\upkappa$B) transcription factor. NF-$\upkappa$B dynamics affects cell fate through the action of dimeric transcription factors that regulate immune responses, cell proliferation and apoptosis (Hayden and Ghosh 2008). In unstimulated cells NF-$\upkappa$B is sequestered in the cytoplasm by association with the inhibitor kappa B (I$\upkappa$B) family of proteins. Upon stimulation with cytokines, such as tumour necrosis factor $\alpha$ (TNF$\upalpha$), the I$\upkappa$Bs are degraded releasing NF-$\upkappa$B to the nucleus where it activates the transcription of over 300 target genes (Hoffmann and Baltimore 2006). Single cell fluorescence imaging has shown that upon continuous TNF$\upalpha$ stimulation NF-$\upkappa$B exhibits nuclear-to-cytoplasmic oscillations with a period of approximately 100 min (Nelson et al. 2004). This period is critical for maintaining downstream gene expression (Ashall et al. 2009). The oscillatory dynamics emerge through the interplay of a number of negative and positive feedback genes that are under the transcription control of NF-$\upkappa$B. These, among others, include the I$\upkappa$B and A20 inhibitors, and cytokines such as TNF$\upalpha$ (Fig. 1) (Hoffmann and Baltimore 2006). In order to understand this intricate feedback regulation various mathematical models of the NF-$\upkappa$B signalling network have been proposed (Hoffmann et al. 2002; Lipniacki et al. 2004; Mengel et al. 2012; Turner et al. 2010). However, the overall system is not fully resolved.

The large number of variables and biochemical reactions in dynamic models, such as those of the NF-$\upkappa$B system, makes them analytically intractable. Sensitivity analyses are often employed to understand these models, assessing how individual parameters influence model dynamics in a local and global context (Ihekwaba et al. 2004, 2005; Rand 2008). Model reduction approaches provide a complementary avenue to extract the core reactions and variables responsible for the key dynamical features of the system. These include modularisation to break large systems down into more tractable functional units (Saez-Rodriguez et al. 2004). However, definition of a module becomes arbitrary, so this remains a heuristic technique. Other techniques include using a posterori analysis and characteristic timescales. Based on error analysis, the former method identifies, for different time intervals, the components of the model required for accurate representation of the solution and uses this information to guide model simplification (Whiteley 2010). The latter utilises the fact that many biological systems incorporate markedly different time-scales ranging from seconds to hours. Relevant approaches employ the use of partial-equilibriums (PE), quasi-steady-state approximations (QSSA), or grouping variables with equivalent time-scales (Krishna et al. 2006; Maeda et al. 1998; Schneider and Wilhelm 2000), see also Kutumova et al. (2013) and Radulescu et al. (2008) for analysis of the NF-$\upkappa$B signalling. These methods often rely on intuition to identify the small parameters that allow the successive reduction steps, and a standard problem for perturbation methods is that in reality the small parameters are never infinitely small and one needs somehow to assess whether they are small enough for any particular purpose, that is, additional accuracy control is required. Algorithmic approaches to identification of small parameters have been proposed. For instance, Computational Singular Perturbation (CSP) method is an iterative procedure, based on identification of the fast modes through the analysis of the eigenvalues of the Jacobian matrix (Lam and Goussis 1994), see also Kourdis et al. (2013) for the asymptotic analysis of the NF-$\upkappa$B dynamics. Other methods exploiting the eigenvalues of the Jacobian are the Intrinsic Low-Dimensional Manifolds (ILDM) method by Maas and Pope (1992) and a more refined Method of Invariant Manifold by Gorban and Karlin (2003). Comparison and analysis of these methods can be found in (Zagaris et al. 2004). Although these methods are more advanced that the classical PE and QSSA techniques, they are also more technically challenging than their predecessors. QSSA methods retain original variables and parameters. Alternative methods, such as the Elimination of Nonessential Variables (ENVA) method described in Danø et al. (2006) exploit searches through lower-dimensional models of reduced networks for a minimal mathematical model which will reproduce a desired dynamic behaviour of the full model. Such a systematic reduction method has the advantage of requiring neither knowledge of the minimal structures, nor re-parameterisation of the retained lumped model components. Indeed, application of model reduction methods which are algorithmic rather than necessarily biologically intuitive can clearly reveal model sub-structures which control basic system dynamics.

In this manuscript we use a simple algorithmic QSSA approach for the reduction of biochemical reaction systems using a heuristic that is likely to be widely applicable to this sort of systems. We define “speed coefficients” that enable ranking variables according to how quickly their approach their momentary steady-state. This allows a straightforward choice of variables for elimination by QSSA application at each step of the algorithm, while preserving dynamic characteristics of the system. We use this method to derive reduced models of the NF-$\upkappa$B signalling network. Our analysis identifies the key feedback components of the system responsible for NF-$\upkappa$B dynamics. Further, reduction of the NF-$\upkappa$B model around different solutions (corresponding to different experimental protocols) revealed specific components of the IKK signalling module responsible for generation of the respective dynamics. This demonstrates the application of an essentially local technique which can be used to infer information about the system in a larger context, ultimately providing a better understanding of the NF-$\upkappa$B signalling network.

## Methods

### Perturbation theory for fast-slow systems

The application of steady-state approximation to biochemical reaction systems typically argues that some of the reagents are highly reactive, so are used as quickly as they are made. Therefore, after the initial transient phase, the concentration of such a reagent is always close to what would be its steady-state as long as concentrations of other reagents were maintained constant. In the simplest form, this means that in the kinetic equations, the corresponding rate of change can be set to zero. This provides a general procedure for simplifying biochemical systems, based on the difference of characteristic time-scales. Practical application of this idea dates back at least to Briggs and Haldane (1925). More recent reviews and textbook expositions can be found (e.g. in Klonowski 1983; Segel and Slemrod 1989; Volpert and Hudjaev 1985; Yablonskii et al. 1991). The basic mathematical justification of the formal procedures stems from the seminal work by Tikhonov (1952). It is formulated for systems which involve small parameter $\varepsilon$ in the form

1 $$\begin{aligned} \frac{\mathrm {d}{x}}{\mathrm {d}{t}}&= f\left( x,z,t\right) ,\nonumber \\ \varepsilon \frac{\mathrm {d}{z}}{\mathrm {d}{t}}&= g\left( x,z,t\right) , \end{aligned}$$

where $x$ is a vector of slow variables and $z$ is the vector of fast variables. In the limit $\varepsilon \rightarrow 0$, the system (1) becomes

2 $$\begin{aligned} \frac{\mathrm {d}{x}}{\mathrm {d}{t}}&= f\left( x,z,t\right) ,\nonumber \\ z&= \phi \left( x,t\right) , \end{aligned}$$

where $\phi \left( x,t\right)$ is the solution of $g\left( x,z,t\right) =0$. If $\varepsilon$ is small, the solutions to the original system (1) may be expected to differ from solutions of (2) only slightly. For an initial-value problem for a finite time interval this is guaranteed by the following:

#### **Theorem 1**

Let the right-hand sides of systems (1) and (2) be sufficiently smooth so solutions to initial value problems exist and are unique. Let $x=X(t;\varepsilon )$, $z=Z(t;\varepsilon )$, $t\in [0,T]$, $T>0$ be a solution of the system (1) with initial condition $X(0;\varepsilon )=x_0$, $Z(0;\varepsilon )=z_0$, and $x=\bar{X}(t)$ be a solution to the system (2) with initial condition $\bar{X}(0)=x_0$. Consider also the “attached” system,

3 $$\begin{aligned} \frac{\mathrm {d}{z}}{\mathrm {d}{s}} = g\left( x,z,t\right) , \end{aligned}$$

depending on $x$ and $t$ as parameters. Let $z=\phi (x,t)$ be a function defined on an open set containing the trajectory $\{(\bar{X}(t),t), t\in [0,T]\}$, such that $z=\phi (\bar{X}(t),t)$ is an isolated, Lyapunov stable and asymptotically stable equilibrium of (3) for the corresponding $x=\bar{X}(t)$ and any $t\in [0,T]$. Finally, assume that $z_0$ is within the basin of attraction of the equilibrium $\phi (x_0,0)$ of system (3) at $x=x_0$, $t=0$. Then for any $t\in (0,T]$,

$$\begin{aligned} {\mathop {\lim }\limits _{\varepsilon \rightarrow 0}} X(t;\varepsilon ) = \bar{X}(t), \qquad {\mathop {\lim }\limits _{\varepsilon \rightarrow 0}} Z(t;\varepsilon ) = \phi (\bar{X}(t)). \end{aligned}$$

This theorem is a special case of Theorem 1 of  Tikhonov (1952). In fact, the solution of the full system (1) can be considered as consisting of two parts: the initial transient, approximately described by (3), with $s=\varepsilon t$, and $x\approx x_0$, which is followed by the long-term part, approximately described by the solution $x=\bar{X}(t)$, $z=\phi (x)$. However the duration of the transient is $\fancyscript{O}\left( \varepsilon \right)$ so for any fixed $t>0$ and sufficiently small $\varepsilon$, the initial transient will have expired by the time $t$, hence the limit.

A limitation of the above result is that it gives only pointwise convergence in $\varepsilon$ so it does not answer the questions about the behaviour of trajectories as $t\rightarrow 0$ at a fixed $\varepsilon$. There were later extensions of this work, relieving this limitation. In this paper we will be looking at periodic solutions, so the following result is relevant to us:

#### **Theorem 2**

In addition to the assumptions of Theorem 1, suppose that the slow system (2) has a periodic solution with period $P_0$, that is $x=\tilde{X}(t)$: $\tilde{X}(t+P_0)\equiv \tilde{X}(t)$, and this solution is stable in the linear approximation. Then the full systems (1) have an ($\varepsilon$-dependent) family of periodic solutions with periods $P(\varepsilon )$ such that $\lim _{\varepsilon \rightarrow 0}P(\varepsilon )=P_0$ and the corresponding orbits lie in a small vicinity of $(\tilde{X}(t),\phi (\tilde{X}(t)))$ for small $\varepsilon$. Moreover, the periodic orbits and the period depend of $\varepsilon$ smoothly.

This theorem is a special case of Theorem 5 of Anosov (1960).

When the approximation of the solution of the full system by that of the slow system is insufficient in itself, it can be improved by considering higher-order corrections in $\varepsilon$. The mathematical justification of that procedure is based on the results about smoothness of the dependence of solutions of the full system on $\varepsilon$, see e.g. Vasil’eva (1952). A very influential continuation of these works with important generalizations, under a currently popular name of “geometric perturbation theory”, has been done by Fenichel (1979). Below we present a simple illustration of the method, directly applicable to our situation.

### Identification of small parameters: parametric embedding

In the real-life kinetic equations it is not always obvious which reagents can be suitable for the QSSA. To identify such reagents, we follow the formal method of “parametric embedding” (Suckley and Biktashev 2003; Biktasheva et al. 2006).

#### **Definition 1**

We will call a system

$$\begin{aligned} \dot{u} = F(u;\varepsilon ), \qquad u\in \mathbb {R}^{d}, \end{aligned}$$

depending on parameter $\varepsilon$, a *1-parametric embedding* of a system

$$\begin{aligned} \dot{u} = f(u), \qquad u\in \mathbb {R}^{d}, \end{aligned}$$

if $f(u)\equiv F(u,1)$ for all $u\in \mathop {\mathrm {dom}}\left( f\right)$. If the limit $\varepsilon \rightarrow 0$ is concerned then we call it a *asymptotic embedding*. If a 1-parametric embedding has a form (1), we call it a *Tikhonov embedding*.

The typical use of this procedure has the form of a replacement of a small constant with a small parameter. If a system contains a dimensionless constant $a$ which is “much smaller than 1”, then replacement of $a$ with $\varepsilon a$ constitutes a 1-parametric embedding; and then the limit $\varepsilon \rightarrow 0$ can be considered. In practice, constant $a$ would more often be replaced with parameter $\varepsilon$, but mathematically, in the context of $\varepsilon \rightarrow 0$ and $a=\mathrm {const}\ne 0$ this, of course, does not make any difference from $\varepsilon a$. This explains the paradoxical use of a zero limit for a parameter whose true value is one.

In some applications, the “small parameters” appear naturally and are readily identified. However, this is not always the case, and in complex nonlinear systems asymptotic analysis may require this procedure of parametric embedding, i.e. introduction of small parameters artificially. It is important to understand, that there are infinitely many ways a given system can be parametrically embedded, as there are infinitely many ways to draw a curve $F(u;\varepsilon )$ in the functional space given the only constraint that it passes through a given point, $F(u;1)=f(u)$. In terms of asymptotics, which of the embeddings is “better” depends on the qualitative features of the original systems that need to be represented, or classes of solutions that need to be approximated. Some examples of different Tikhonov embeddings of a simple cardiac excitation model can be found in Suckley and Biktashev (2003), and non-Tikhonov embedding of the same in Biktashev and Suckley (2004), and some of those examples are better than others in describing particularly interesting features of cardiac action potentials.

If a numerical solution of the system can be found easily, then there is a simple practical recipe: to look at the solutions of the embedding at different, progressively decreasing values of the artificial small parameter $\varepsilon$, and see when the features of interest will start to converge. If the convergent behaviour is satisfactorily similar to the original system with $\varepsilon =1$, the embedding is adequate for these features.

To summarize, we claim that identification of small parameters in a given mathematical model with experimentally measured functions and constants will, from the formal mathematical viewpoint, always be arbitrary (even though in the simplest cases there may be such a natural choice that this ambiguity is not even realized by the modeller), and “validity” of such identification can be defined only empirically: if the asymptotics describe the required class of solutions sufficiently well. The rare exceptions are when the asymptotic series are in fact convergent and the residual terms can be estimated a priori. A cruder (and less reliable) estimate of the error of an asymptotic can be obtained through the analysis of the higher-order asymptotics, see e.g. Turányi et al. (1993); more about it later.

In this paper, we restrict consideration to Tikhonov embeddings (1). The simplest version of the above recipe results in the straightforward procedure: compare the solution of the full system with the solution where the putative fast variable has been replaced by its quasistationary value. In terms of the “numerical embedding”, this means a short-cut: considering values $\varepsilon =1$ and $\varepsilon =0$ instead of a (or as a very short) sequence of values of $\varepsilon$ converging to 0. Although sometimes we have indeed studied several values of $\varepsilon$, we shall always present only $\varepsilon =1$ and $\varepsilon =0$ results, to avoid cluttering the graphs.

### Speed coefficients

It follows from the above discussion that the “numerical embedding” procedure could be applied to any of the dynamic variables, and those whose adiabatic elimination would cause the smallest changes in the solution, could be taken as the fastest. In practice, for a large system, this exhaustive trial and error procedure may be too laborious. We employ a simple heuristic method to identify the candidates for the fastest variables.

We describe it in terms of a generic system of $N$ ordinary differential equations (ODEs),

4 $$\begin{aligned} \frac{\mathrm {d}{x_i}}{\mathrm {d}{t}} = f_{i}\left( x_{1},\dots , x_{N}\right) , \qquad i=1,\dots N. \end{aligned}$$

We define the “speed coefficients” for each dynamic variable $x_i$ as

5 $$\begin{aligned} \lambda _i \left( x_{1},\ldots ,x_{N}\right) = \left| \frac{\partial {f_i}}{\partial {x_i}}\right| . \end{aligned}$$

**Fig. 2:**
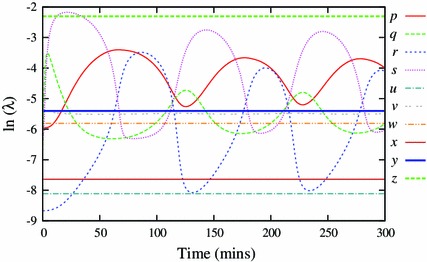
Semi-logarithmic plot of speed coefficients for the Simplified Model (SM). A larger speed coefficient means the variable is approaching its steady state faster. These coefficients identify variable $z$ as the fastest, and therefore the most appropriate candidate for elimination

By definition, these coefficients depend on the dynamic variables, or, for a selected solution, they depend on time $t$. They can be used to rank the variables according to how quickly they approach their momentary steady-states (Fig. 2).

It is very essential to understand that with the exception of relatively trivial cases, the most adequate choice of embedding will depend on the type of solutions that are of interest for the particular application at hand, because in a nonlinear system, what is “small” and what is “large” may be significantly different in different parts of the phase space. A simple but very instructive example illustrating this point is considered by Lam and Goussis (1994, Section A), where the meaning of fast/slow changes depending on initial conditions and on what part of the solution is considered. Our practical approach is that we start from one particular solution, which is selected in such a way that to be sufficiently representative for the class of solutions that are of interest to a particular application. An obvious extension would be selection of a representative set of solutions; however for the illustration of the method, one is enough. As follows from the above, the task of selecting such solutions is inevitably the responsibility of the investigator who is going to apply the method and use the resulting reduced system. In the particular models we consider here this task is relatively straightforward, as the long-term behaviour is more or less the same for any physiologically sensible initial conditions. For elimination of any further ambiguity we have adopted a rule that we would select for elimination the variable that is fastest at its slowest. That is, for each variable we find the minimal value of its speed coefficient over the simulated time interval, and then select the variable which has the highest value of the minimal speed among other variables.

Note that our heuristic procedure only uses partial information about the system (only the diagonal elements of the Jacobian, and only its minimal value along only one/a few solution(s)), but it is only used for preliminary selection of variables for reduction. Therefore, the actual success of reduction is established by comparison of the reduced and the original system, within the “numerical embedding” procedure described above. In the test cases presented in this paper, this proof has always been successful, if sometimes with first-order corrections. However one cannot exclude the possibility that high relative values of the non-diagonal elements of the Jacobian and/or its strong variations over the representative solutions may force the change of the candidate for reduction, or QSSA may be inapplicable in principle. As an extreme example, consider a subsystem: $\dot{x}=Ay$, $\dot{y}=-Ax$, which has zero diagonal Jacobian elements, so would be classed as (infinitely) slow, yet for large $A$ its treatment as such within a larger system would produce wrong results, as in fact $x$ and $y$ will fastly oscillate. For the (bio-)chemical kinetics this sort of behaviour is, however, not very likely, at least at the level of elementary reactions; see e.g. the discussion in Turányi et al. (1993, p. 165). On the other hand, this fastly oscillating subsystem is not appropriate for Tikhonov style treatment anyway, and requires averaging in Krylov–Bogolyubov style instead; whereas if a system *does* have the form (1) and satisfies the assumptions of Theorem 1, then the eigenvalues of the Jacobian block $\varepsilon ^{-1}\partial {g}/\partial {z}$ have negative real parts and are of the order of $\varepsilon ^{-1}$, so its diagonal elements are likely to be large (and negative)—although, of course, counter-examples can be invented.

Finally, we note again that the choice of variables for reduction may depend on the class of solutions of interest, which in our approach will be done via the choice of representative solution. In Sects. 3 and 4 we consider two different classes of solution in the same full model, which give two different reduced models.

### The model reduction algorithm

Based on Tikhonov’s and Anosov’s theorems and the definition of the speed coefficients we can define a general method for reducing the dimension of a biochemical reaction system. We illustrate the method using an example where the right-hand side of an ordinary differential equation for a fast variable is linear with respect to the same variable. Suppose the variable $x_j$ has been identified as the fast variable in the system (4). With account of the artificial small parameter, this gives

6 $$\begin{aligned} \varepsilon \frac{\mathrm {d}{x_j}}{\mathrm {d}{t}}=\alpha _j(t)-\beta _j(t)x_j, \end{aligned}$$

where coefficients $\alpha _j(t)$ and $\beta _j(t)$ are presumed to depend on time via other dynamic variables. We look for a solution in the form of an asymptotic series $x_j=x_j^0+\varepsilon x_j^ 1+\varepsilon ^2x_j^ 2+\fancyscript{O}\left( \varepsilon ^3\right)$. Substituting this into (6) gives

7 $$\begin{aligned} \varepsilon \dot{x}_j^0+\varepsilon ^2\dot{x}_j^1+\varepsilon ^3\dot{x}_j^2 = \alpha _j-\beta _j x_j^0-\varepsilon \beta _j x_j^1-\varepsilon ^2\beta _j x_j^2 + \fancyscript{O}\left( \varepsilon ^{3}\right) . \end{aligned}$$

The simplest approximation for $x_j$ is obtained by considering the terms in (7) proportional to $\varepsilon ^0$,

8 $$\begin{aligned} 0 = \alpha _j(t)-\beta _j(t)x^0_j, \end{aligned}$$

which results in the zeroth-order QSSA for variable $x_j$:

9 $$\begin{aligned} \overline{x}_j^{0} = x_j^{0}=\frac{\alpha _j(t)}{\beta _j(t)}. \end{aligned}$$

This approximation $x_j^0$ is then substituted into the original system of equations for the variable $x_j$. If the variable is sufficiently fast then this steady-state expression should be a good approximation of the fast variable and the substitution will cause minimal change to the solution.

In general, the zeroth-order QSSA provides a reasonable approximation of the original variable. However, if such approximation is not good enough, it can be improved by calculating an additional correction term. To do this we consider terms in (7) proportional to $\varepsilon ^1$:

10 $$\begin{aligned} \varepsilon \dot{x}_j^0 = -\varepsilon \beta _j x_j^1. \end{aligned}$$

Substituting our earlier result (9) into Eq. (10) and solving for $x_j^{1}$ gives the first-order correction in the form

11 $$\begin{aligned} x_j^1 = -\frac{1}{\beta _j}\dot{x}_j^0 = \frac{\alpha _j\dot{\beta _j}-\beta _j\dot{\alpha _j}}{\beta _j^{3}}. \end{aligned}$$

This results in the first-order QSSA $\overline{x}_j^{1}=x_j^0+\varepsilon x_j^1$ in the form

12 $$\begin{aligned} \overline{x}_j^{1} = \frac{\alpha _j(t)}{\beta _j(t)} + \frac{ \alpha _j(t)\dot{\beta _j}(t)-\beta _j(t)\dot{\alpha _j}(t) }{ \beta _j^{3}(t) }, \end{aligned}$$

since the original problem corresponds to $\varepsilon =1$. Note that the value of the first-order correction, or its estimate, can be used as an estimate of the accuracy of the leading-term approximation; roughly speaking, this is the idea behind the accuracy estimate used in Turányi et al. (1993).

So our method can then be formulated into a general algorithm to reduce the dimension of a biochemical system defined by ordinary differential equations. The algorithm reads:

1. Using numerical methods, find a representative solution of the system of ODEs for the chosen time interval.
2. Calculate the expressions for the speed coefficients ($\lambda$’s), using Eq. (5) from the system of ODEs (this can be assisted by a symbolic calculations software, e.g. Maple).
3. Substitute the numerical solution of the system into the expressions for the $\lambda$’s to find the speed for each variable at each time point.
4. Plot the speed coefficients vs. time and identify the fastest variable (at its slowest).
5. Calculate the expression for the zeroth-order QSSA using (9).
6. Substitute this QSSA into the system of ODEs to eliminate the fastest variable, thus obtaining a reduced system.
7. Compare the solution of the reduced system with the solution of the original system.
8. *If the zeroth-order QSSA is insufficient to maintain a suitable accuracy, calculate the first-order QSSA using equation* (12).
9. Repeat the above process for the new reduced system.

## Minimal model of the NF-$\upkappa$B system in response to continuous TNF$\upalpha$ input

The “two-feedback” model of the NF-$\upkappa$B system presented in Ashall et al. (2009) is our starting point. It is a system of 14 ordinary differential equations representing NF-$\upkappa$B and the I$\upkappa$B$\upalpha$ and A20 negative feedbacks (Fig. 1). We use brief notations for its variables and parameters as given in Table 1. We pursued derivation of a minimal model with respect to a representative solution obtained for initial conditions as described in Table 1 and $k_{24}=1$. In a biological context this corresponds to a continuous stimulation of the system with a high dose of TNF$\upalpha$ (Ashall et al. 2009).

**Table 1:** Variables and parameters: their names as in Ashall et al. (2009), short names adopted here, and values

Variables ($\upmu \mathrm {M}$)			Parameters			Parameters		
$NF\upkappa B$	$p$	$3.81\times 10^{-3}$	$kv$	$k_1$	$3.3$	$ki1$	$k_{15}$	$0.0026\,\mathrm {s}^{-1}$
$I\upkappa B\alpha$	$q$	$1.58\times 10^{-2}$	IKK$^\mathrm{a}$	$k_2$	$0.08\,\upmu \mathrm {M}$	$ke1$	$k_{16}$	$0.000052\,\mathrm {s}^{-1}$
$nNF\upkappa B$	$r$	$9.79\times 10^{-3}$	NF-$\upkappa$B$^\mathrm{b}$	$k_3$	$0.08\,\upmu \mathrm {M}$	$kc1a$	$k_{17}$	$0.074\,\mathrm {s}^{-1}$
$nI\upkappa B\alpha$	$s$	$5.44\times 10^{-3}$	$ka1a$	$k_4$	$0.5\,(\upmu \mathrm {M}\,\mathrm {s})^{-1}$	$kc2a$	$k_{18}$	$0.37\,\mathrm {s}^{-1}$
$tI\upkappa B\alpha$	$u$	$2.07\times 10^{-5}$	$c1a$	$k_5$	$1.4\times 10^{-7}\,(\upmu \mathrm {M}\,\mathrm {s})^{-1}$	$kt2a$	$k_{19}$	$0.1\,\mathrm {s}^{-1}$
$IKKn$	$v$	$0.08$	$c2a$	$k_6$	$0.5\,\mathrm {s}^{-1}$	$kp$	$k_{20}$	$0.0006\,\mathrm {s}^{-1}$
$IKKa$	$w$	$0$	$c3a$	$k_7$	$0.0003\,\mathrm {s}^{-1}$	$kbA20$	$k_{21}$	$0.0018\,\upmu \mathrm {M}$
$tA20$	$x$	$6.46\times 10^{-6}$	$c4a$	$k_8$	$0.0005\,\mathrm {s}^{-1}$	$ka$	$k_{22}$	$0.004\,\mathrm {s}^{-1}$
$A20$	$y$	$7.19\times 10^{-4}$	$c1$	$k_9$	$1.4\times 10^{-7}(\upmu \mathrm {M}\,\mathrm {s})^{-1}$	$ki$	$k_{23}$	$0.003\,\mathrm {s}^{-1}$
$pI\upkappa B\alpha \circ NF\upkappa B$	$z$	$0$	$c2$	$k_{10}$	$0.5\,\mathrm {s}^{-1}$	$TR$	$k_{24}$	$1/0$
$IKKi$	$a$	$0$	$c3$	$k_{11}$	$0.00048\,\mathrm {s}^{-1}$	$k$	$k$	$0.065\,\upmu \mathrm {M}$
$pI\upkappa B\alpha$	$b$	$0$	$c4$	$k_{12}$	$0.0045\,\mathrm {s}^{-1}$	$h$	$h$	$2$
$nI\upkappa B\alpha \circ nNF\upkappa B$	$c$	$7.95\times 10^{-4}$	$ki3a$	$k_{13}$	$0.00067\,\mathrm {s}^{-1}$			
$I\upkappa B\alpha \circ NF\upkappa B$	$d$	$7.30\times 10^{-2}$	$ke3a$	$k_{14}$	$3.35\times 10^{-4}\,\mathrm {s}^{-1}$			

The initial conditions were obtained by equilibrating the system without stimulus ($k_{24}=0$), with $v$ and $r$ set to $k_2$ and $k_3k_1$, respectively, and other variables set to 0

$^\mathrm{a}$ IKK$=v+w+a$ (conserved quantity)

$^\mathrm{b}$NF-$\upkappa$B$=p+d+z+\frac{1}{k_1}(r+c)$ (conserved quantity)

Before employing the reduction algorithm we endeavoured to simplify the system by elementary means (similarly to Wang et al. 2012). Conservation of cellular IKK reads $v+w+a=k_2=\mathrm {const}$, which allows us to eliminate $a$ via the substitution $a=k_2-v-w$. Similarly, conservation of NF-$\upkappa$B in all its five forms reads $p+d+z+\frac{1}{k_1}(r+c)=k_3=\mathrm {const}$, which we use to eliminate $d$. Further, we observed that variable $b$ is “decoupled”: it is only present in its own equation, and the dynamics of other variables do not depend on it. So it can be removed from the analysis, as the solution for it, if necessary, can be obtained post factum by integration of the solution of the remaining system. Finally, for this representative solution we have observed that some of the terms in the equation consistently remain so small that their elimination does not visibly change the solution. This involved elimination of variable $c$, leaving a system of ten equations, which we shall refer to as the Simplified Model (SM):

13a $$\begin{aligned} \frac{\mathrm {d}{p}}{\mathrm {d}{t}}&= k_{19}z-k_4qp-k_{15}p+k_{16}r\end{aligned}$$

13b $$\begin{aligned} \frac{\mathrm {d}{q}}{\mathrm {d}{t}}&= -k_4qp+k_6u-k_8q-k_{13}q+k_{14}s-k_{17}wq\end{aligned}$$

13c $$\begin{aligned} \frac{\mathrm {d}{r}}{\mathrm {d}{t}}&= k_{15}k_1p-k_4sr-k_{16}k_1r\end{aligned}$$

13d $$\begin{aligned} \frac{\mathrm {d}{s}}{\mathrm {d}{t}}&= k_{13}k_1q-k_4sr-k_8s-k_{14}k_1s\end{aligned}$$

13e $$\begin{aligned} \frac{\mathrm {d}{u}}{\mathrm {d}{t}}&= k_5\frac{r^{h}}{r^{h}+k^{h}}-k_7u\end{aligned}$$

13f $$\begin{aligned} \frac{\mathrm {d}{v}}{\mathrm {d}{t}}&= k_{20}\frac{k_{21}}{k_{21}+k_{24}y}\left( k_2-v\right) -k_{24}k_{22}v\end{aligned}$$

13g $$\begin{aligned} \frac{\mathrm {d}{w}}{\mathrm {d}{t}}&= k_{24}k_{22}v-k_{23}w\end{aligned}$$

13h $$\begin{aligned} \frac{\mathrm {d}{x}}{\mathrm {d}{t}}&= k_9\frac{r^{h}}{r^{h}+k^{h}}-k_{11}x\end{aligned}$$

13i $$\begin{aligned} \frac{\mathrm {d}{y}}{\mathrm {d}{t}}&= k_{10}x-k_{12}y\end{aligned}$$

13j $$\begin{aligned} \frac{\mathrm {d}{z}}{\mathrm {d}{t}}&= k_{18}w\left( k_3-p-\frac{r}{k_1}\right) -k_{19}z\end{aligned}$$

The solution of (13) is very close to that of the original model (see Table 2 and Appendix A), and marks the starting point of the reduction procedure. We apply the reduction algorithm iteratively, eliminating a sequence of fast variables and employing different orders of approximation for them. To keep track of these, we introduce a nomenclature for the reduced models. The model variants are named according to the variables that have been removed, each with a subscript showing if a zeroth- or first-order QSSA has been used, $0$ or $1$ respectively. For example, the first variable eliminated is $z$, therefore the model with this variable replaced with a zeroth-order QSSA is titled $z_0$ and the same with a first-order QSSA is titled $z_1$. A model where the variables $z$ and $p$ have been replaced in turn with their zeroth- and first-order QSSAs respectively, will be denoted as $z_0p_1$, etc. Below, we concentrate on the key points of the reduction sequence.

**Table 2:** Key features of NF-$\upkappa$B oscillations for each of the model variants

Model	Variable removed at this stage	Period (mins)	Fold change in period	Fold change in amplitude	Shape MSE $\times 10^5$
Original model	N/A	99.5	1	1	N/A
Simplified model	$a,b,c,d$	100.5	1.01	1.06	1.24
$z_0$	$z$	100.0	1.01	1.06	1.05
$z_0p_0$	$p$	92.5	0.93	0.92	2.47
$z_0p_0y_0$	$y$	85.5	0.86	0.83	9.42
$z_0p_0y_0v_0$	$v$	77.7	0.78	0.65	32.4
$z_0p_0y_0v_0s_0$	$s$	75.0	0.75	0.62	38.6
$z_0p_0y_0v_0s_0w_1$	$w$	73.8	0.74	0.71	22.3
$z_0p_1y_0v_0s_0w_1$	As above	80.3	0.80	0.90	5.28
$z_0p_1y_1v_1s_1w_1$	As above	86.6	0.87	1.01	6.31

Fold change in period and amplitude was calculated relative to the period and amplitude of the original model in (Ashall et al. 2009). MSE was calculated after the models had been scaled to have the same period

**Fig. 3:**
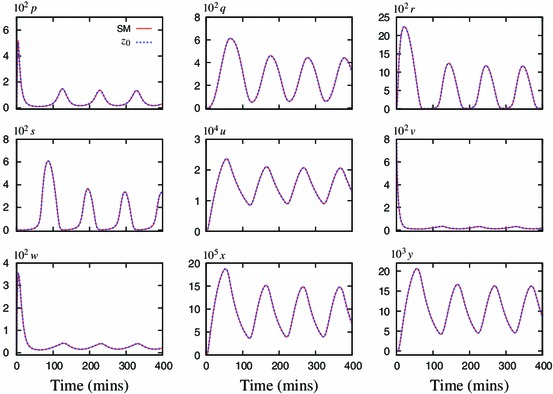
Components of the representative solution for the 10-variable Simplified Model (SM, *solid lines*) and reduced 9-variable model $z_0$ (*dashed lines*). The lines visually coincide in all cases, indicating that zeroth-order approximation is sufficient for $z$

Figure 2 shows the speed coefficients calculated for the Simplified Model. It identifies $z$ to be the fastest and thus eliminated first. Application of the method, using zeroth-order approximation, results in a 9-variable model $z_0$ with comparable solution to this of the Simplified Model (Fig. 3).

**Fig. 4:**
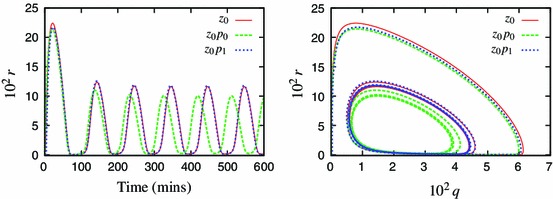
Comparison of the representative solution for the 9-variable model $z_0$ (*solid lines*) and its two 8-variable reductions, with the zeroth-order (*dashed lines*) and the first-order (*dotted lines*) approximations for $p$. *Left panel* shows a solution for the variable nuclear NF-$\upkappa$B, $r$, *right panel* shows a phase plane for the variables $q$ and $r$. Use of the first-order approximation gives a marked improvement in accuracy of the reduced model

Addition of a first-order correction to some of the QSSAs improved the model fit in comparison to respective predecessors. Figure 4 shows that a first-order correction in the variable $p$ markedly improved the accuracy of the 8-variable reduced model. However, addition of these corrections can also increase the algebraic complexity of the system and it must be considered whether the improvement of the model outweighs the added complexity.

**Fig. 5:**
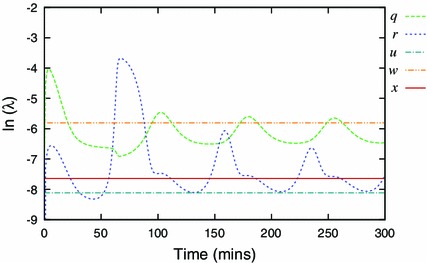
Semi-logarithmic plot of speed coefficients for dynamic variables of the $z_0p_{0}y_{0}v_{0}s_{0}$ model. The variable $w$ has the largest minimum compared to other variables, identifying it as the most appropriate candidate for the next elimination

As the reduction progressed, there was an increasing overlap in the ranges of the speed coefficients, and we had to apply the “the fastest at its slowest” heuristic rule. For example in Fig. 5, this rule identifies the variable $w$ for elimination during reduction to the 4-variable model, even though two other variables, $r$ and $q$, are at times faster.

**Fig. 6:**
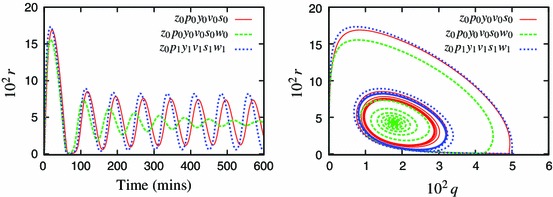
Comparison of the representative solution for the 5-variable model $z_0p_0y_0v_0s_0$ (*solid lines*) and its two possible 4-variable reductions, with the zeroth-order (*dashed lines*) and the first-order (*dotted lines*) approximations for $w$. Use of the first-order approximation not only improved the accuracy of the 4-variable model, but also maintained a stable limit cycle

Successive cycles of the algorithm were applied to ultimately reduce this system to four differential equations. The method maintained the important qualitative features of the system, such as the limit cycle. However, through each stage of the reduction, the resulting limit cycle had a slightly reduced period and amplitude (Table 2). Using only the zeroth-order QSSAs was sufficient to reduce the model to five ODEs ($z_0p_0y_0v_0s_0$), while maintaining the limit cycle. In order to reduce the system further, the use of a first-order QSSA was necessary (Fig. 6). A suitable zeroth- or first-order QSSA could not be calculated to reduce the model beyond this, and therefore the model $z_0p_0y_0v_0s_0w_1$ of four differential equations was chosen as the end point of this analysis. This minimal model is given by  (14), where $A= k_{24}k_{22}k_{20}k_{21}k_{12}k_3$ and $B(x)=k_{20}k_{21}k_{12}+k_{24}k_{22}k_{21}k_{12}+k_{24}^{2}k_{22}k_{10}k_{23}x$.

14a $$\begin{aligned} \frac{\mathrm {d}{q}}{\mathrm {d}{t}}&= -k_4q\bar{p}(q,r,x) +k_6u-k_8q-k_{13}q\nonumber \\&+\,\,k_{14}\bar{s}(q,r) -k_{17}\bar{w}(r,x)q\end{aligned}$$

14b $$\begin{aligned} \frac{\mathrm {d}{r}}{\mathrm {d}{t}}&= k_{15}k_1\bar{p}(q,r,x) -k_4\bar{s}(q,r)r-k_{16}k_1r\end{aligned}$$

14c $$\begin{aligned} \frac{\mathrm {d}{u}}{\mathrm {d}{t}}&= k_5\frac{r^{h}}{r^{h}+k^{h}} -k_7u\end{aligned}$$

14d $$\begin{aligned} \frac{\mathrm {d}{x}}{\mathrm {d}{t}}&= k_9\frac{r^{h}}{r^{h}+k^{h}} -k_{11}x\end{aligned}$$

14e $$\begin{aligned} \bar{w}(r,x)&= \frac{ A }{ B(x) } + \frac{ k_{24}^{2}k_{22}k_{10}A\left( \frac{k_9r^{h}}{r^{h}+k^{h}}-k_{11}x\right) }{ B(x)^{2} }\end{aligned}$$

14f $$\begin{aligned} \bar{s}(q,r)&= \frac{k_1k_{13}q}{k_4r+k_1k_{14}+k_8}\end{aligned}$$

14g $$\begin{aligned} \bar{p}(q,r,x)&= \frac{ k_{16}r+k_{18}\bar{w}(r,x)\left( k_3-\frac{r}{k_1}\right) }{ k_4q+k_{15}+k_{18}\bar{w}(r,x) } \end{aligned}$$

**Fig. 7:**
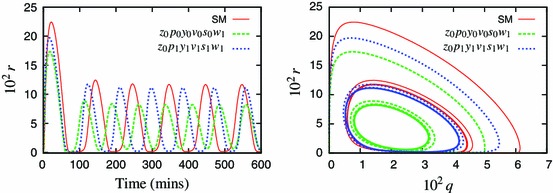
Comparison of the representative solution for the Simplified Model (*solid lines*) and the two four-variable reductions, the cruder $z_0p_0y_0v_0s_0w_1$ model (*dashed lines*) and the more accurate $z_0p_1y_1v_1s_1w_1$ model (*dotted lines*)

It was possible to add first-order corrections to all of the dynamic variables during the model reduction, producing a minimal model $z_1p_1y_1v_1s_1w_1$ with a far improved fit in comparison to the original. However, the $z_0$ approximation was so accurate that $z_1$ did not make a noticeable improvement. Figure 7 shows comparison of the “simplest” and “the most accurate” 4-variable models to the original 10-variable one (the $z_0p_1y_1v_1s_1w_1$ model is presented in Appendix A).

**Fig. 8:**
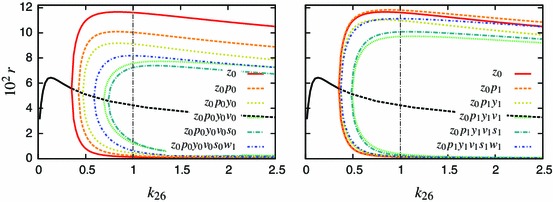
Bifurcation analyses of reduced models with respect to the parameter $k_{24}$, representing the dose of TNF$\upalpha$ stimulation. Branches of the solution in *colour* represent minimal and maximal values of the limit cycle. *Solid* and *dashed black lines* correspond to stable and unstable equilibria, respectively

Figure 8 shows how some of the dynamic properties of the model change through the reduction process. It represents the steady state solution and continuation for the variable $r$ as the parameter $k_{24}$ is varied (Doedel et al. 2000; Ermentrout 2002), showing the effect of altering the TNF$\upalpha$ dose (Turner et al. 2010). In the original model, there is a supercritical Hopf bifurcation (HB) at $k_{24}=0.36$ above which the limit cycle is observed. Successive elimination of the fastest variables causes the HB point to move up, closer to the value $k_{24}=1$, which corresponds to a saturating dose of TNF$\upalpha$. Reduction from five to four differential equations using zeroth-order QSSA for $w$ would move the HB point further to the right (Hopf bifurcation at $k_{24}=3.105$). Figure 8 also demonstrates that use of the first-order correction terms dramatically reduces the loss in limit cycle amplitude and change in the location of the HB point.

## Model reduction with respect to pulsed TNF$\upalpha$ input 

Previously, we derived models with respect a solution that corresponded to a constant value of the TNF$\upalpha$ input, $k_{24}\equiv 1$. The universality of such models depends on how representative that solution actually is. In this subsection we give an example where a different selection of the representative solution leads to a different reduced model.

**Fig. 9:**
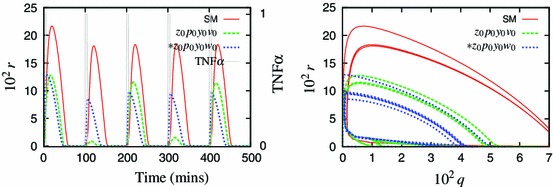
Models’ response to a pulsed TNF$\upalpha$ input. Shown are solution of the Simplified Model (SM, *solid line*), the 6-variable model $z_{0}p_{0}y_{0}v_{0}$ (*dashed line*), and the alternative 6-variable model $*z_{0}p_{0}y_{0}v_{0}$  (15) (*dotted line*). The TNF$\upalpha$ input is varied: $k_{24}=0$ except for 5-min pulses of $k_{24}=1$ delivered every 100 min (shown in *grey lines* on the *left panel*)

We now consider another experimentally relevant case, where the TNF$\upalpha$ input is varied: $k_{24}=0$ except for 5-min pulses of $k_{24}=1$ delivered every 100 min. Under such stimulation, the system exhibits pulses of the nuclear NF-$\upkappa$B entrained to the input frequency (Ashall et al. 2009). Despite the same 100 min period, these pulses are markedly different than oscillations induced with the continuous TNF$\upalpha$ input. The Simplified Model reproduces this property, see Fig. 2 vs. Fig. 9. However, the 6-variable variant, $z_0p_0y_0v_0$ (see Appendix B for equations), does not respond with a full-size nuclear NF-$\upkappa$B translocation to each pulse, and the solution is of a double period, Fig. 9.

**Fig. 10:**
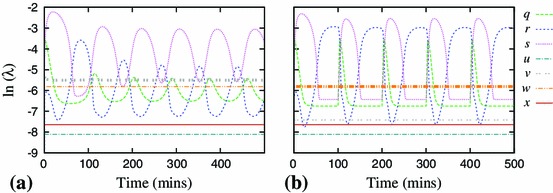
Comparison of the speed coefficients for the $z_0p_0y_0$ calculated with respect to different solutions. **a** Constant input ($k_{24}\equiv 1$). **b** Pulsed input; $k_{24}=0$ except for 5-min pulses of $k_{24}=1$ delivered every 100 min. Depicted in *bold* are the fourth fastest variables: $v$ in **a** and $w$ in **b**

We therefore developed an alternative minimal model, choosing the periodically entrained solution as the representative one. For the periodically entrained solution, the hierarchy of speeds of the variables associated with the IKK module is different from the $k_{24}\equiv 1$ case. Specifically, the first three fastest variables are $z$, $p$ and $y$ as before. However, when choosing the 4th variable for elimination, the neutral form of IKK, $v$, becomes one of the slowest, and the algorithm identified the active IKK, $w$, for approximation (Fig. 10). In the continuous case, $v$ and $w$ were the first and second fastest variables, respectively (Fig. 10). Ultimately, application of the algorithm with respect to the pulsed input resulted in a different model, which showed a much better agreement with the SM and did not display a period doubling (Fig. 9). This alternative 6-variable, ($*z_0p_0y_0w_0$) model is given by:

15a $$\begin{aligned} \frac{\mathrm {d}{q}}{\mathrm {d}{t}}&= -k_4q\bar{p}+k_6u-k_8q-k_{13}q+k_{14}s-k_{17}\bar{w}q\end{aligned}$$

15b $$\begin{aligned} \frac{\mathrm {d}{r}}{\mathrm {d}{t}}&= k_{15}k_1\bar{p}-k_4sr-k_{16}k_1r\end{aligned}$$

15c $$\begin{aligned} \frac{\mathrm {d}{s}}{\mathrm {d}{t}}&= k_{13}k_1\bar{p}-k_4sr-k_8s-k_{14}k_1s\end{aligned}$$

15d $$\begin{aligned} \frac{\mathrm {d}{u}}{\mathrm {d}{t}}&= k_5\frac{r^{h}}{r^{h}+k^{h}}-k_7u\end{aligned}$$

15e $$\begin{aligned} \frac{\mathrm {d}{v}}{\mathrm {d}{t}}&= k_{20}\frac{k_{21}}{k_{21}+k_{24}y}\left( k_2-v\right) -k_{24}k_{22}v\end{aligned}$$

15f $$\begin{aligned} \frac{\mathrm {d}{x}}{\mathrm {d}{t}}&= k_9\frac{r^{h}}{r^{h}+k^{h}}-k_{11}x\end{aligned}$$

15g $$\begin{aligned} \bar{p}&= \frac{k_{16}rk_1+k_{18}\bar{w}k_3k_1-k_{18}\bar{w}r}{k_1\left( k_4q+k_{15}+k_{18}\bar{w}\right) }\end{aligned}$$

15h $$\begin{aligned} \bar{y}&= \frac{k_{10}x}{k_{12}}\end{aligned}$$

15i $$\begin{aligned} \bar{w}&= \frac{k_{24}k_{22}v}{k_{23}} \end{aligned}$$

The difference in the $v$ speed for alternative TNF$\upalpha$ stimulation can be easily understood by analysing the dynamic equation for $v$. The last term in its right-hand side, $-k_{24}k_{22}v$, directly contributes towards decay of $v$, but only when $k_{24}$ is switched on. So when $k_{24}$ is off, the $v$ variable is much slower and its adiabatic elimination is not justified.

## Application of speed coefficients method to Krishna model

Here, we compare the behaviour and properties of two reduced models of Krishna’s full 6-variable model for NF-$\upkappa$B signalling dynamics (Krishna et al. 2006), one obtained by combination of coarse graining and numerical observations, and the other obtained using our new method of speed coefficients. In this analysis, we demonstrate better agreement with the full model achieved using our algorithmic approach.

**Fig. 11:**
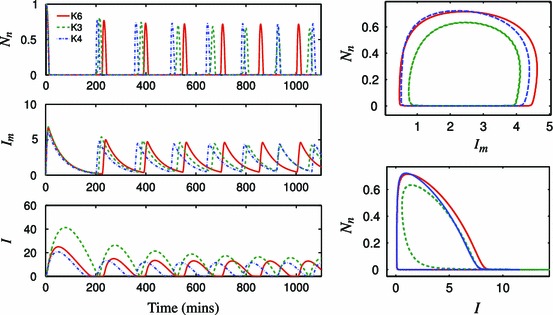
Analysis of alternatively reduced models of the NF-$\upkappa$B system. *Left-hand panel* Time courses for the 3-variable reduced model (K3) and its 6-variable predecessor developed in Krishna et al. (2006) (K6), together with a new 4-variable reduced model obtained using the speed coefficient method (K4). Variables $N_{n},\;I_{m},\;I$ represent nuclear NF-$\upkappa$B, I$\upkappa$B$\upalpha$ protein and I$\upkappa$B$\upalpha$ mRNA respectively. *Right-hand panel* Corresponding phase portraits for the limit cycles that the respective systems approach

Firstly, in Fig. 11, we show time courses for oscillatory solutions for variables representing nuclear NF-$\upkappa$B, I$\upkappa$B$\upalpha$ protein and I$\upkappa$B$\upalpha$ mRNA in three models, namely Krishna’s full model (K6), Krishna’s 3-variable minimal model (K3), and a new 4-variable reduced model given by our speed coefficient algorithm (K4) (see Appendix D for the systems of equations). We note that, while neither of the reduced models matches the full model in period, the oscillation amplitudes of the three variables show reasonable agreement, with our new reduced model (K4) more closely agreeing with the full model. Also, the K4 I$\upkappa$B$\upalpha$ protein profile shape shows better agreement with K6 than K3 does, with $I$ flattening out in its troughs. Summary phase portraits clearly show that K4’s limit cycles more closely agree with K6 than K3 does.

**Fig. 12:**
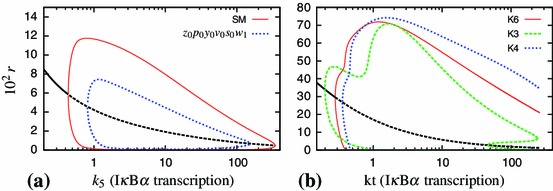
Analysis of alternatively reduced models of the NF-$\upkappa$B system. Shown are bifurcation diagrams with respect to the rate of the I$\upkappa$B$\upalpha$ transcription. Simulations performed for a continuous TNF$\upalpha$ input. **a** The minimal $z_0p_0y_0v_0s_0w_1$ model developed herein (14) in comparison to the SM. The I$\upkappa$B$\upalpha$ transcription rate was normalised to its nominal value (as in Table 1). **b** The 3-variable reduced model (K3) and its 6-variable predecessor developed in Krishna et al. (2006) (K6), together with a new 4-variable reduced model obtained using the speed coefficient method (K4). Over the range of $kt$ shown, the new reduced model gives better qualitative agreement with the full model, and does not introduce the subcritical Hopf bifurcation seen in K3

In Fig. 12 we compare bifurcation diagrams (with respect to the rate of I$\upkappa$B$\upalpha$ transcription) for reduced models with their corresponding full models, for both our Simplified Model and the Krishna model. For the Krishna model, we further compare the Krishna minimal model (K3)and our new reduced model (K4). The reduced model resulting from the speed coefficient method applied to the Simplified Model (SM) gives a bifurcation diagram in qualitative agreement with that for the corresponding full model over the range of $k_{5}$ shown (Fig. 12a). Also, the reduced model resulting from our method applied to the Krishna model (see Appendix D) gives qualitative agreement with the full Krishna model (Fig. 12b). This is a marked improvement over the Krishna minimal model, which demonstrates features that are not present in the corresponding full model. These include variation of the limit cycle amplitude for values of the I$\upkappa$B$\upalpha$ transcription around 1, and a subcritical Hopf bifurcation at around $kt=50$, with unstable limit cycles and hysteresis for the values between 50 and 240. On the contrary, our minimal model preserves the properties of the full model at least at the qualitative level, even for the values of the parameter very different from the one corresponding to the representative solution.

We conclude that application of our method of speed coefficients can produce a reduced model of comparable dimensionality while better preserving the dynamic properties of the original system than other existing techniques.

## Discussion

A key problem in computational and systems biology is to understand how dynamical properties of a system arise via the underlying biochemical networks. However, as these networks involve many components this task becomes analytically intractable and computationally challenging. In this manuscript we present a clearly defined and accessible QSSA algorithm for reduction of such biochemical reaction systems. The method proposed relies on the derivation of speed coefficients to rank system variables according to how quickly they approach their momentary steady state. This enables a systematic method for selection of variables for steady-state approximation at each step of the algorithm.

We used the method to derive a minimal models of the NF-$\upkappa$B signalling network, a key regulator of the immune response (Hayden and Ghosh 2008). Single cell time-lapse analyses showed that the NF-$\upkappa$B system exhibits oscillatory dynamics in response to cytokine stimulation (Nelson et al. 2004; Turner et al. 2010; Tay et al. 2010). It has been shown that the frequency of those oscillations may govern downstream gene expression and therefore be the key functional output of the system (Ashall et al. 2009; Sung et al. 2009; Tay et al. 2010). The ability to control the NF-$\upkappa$B dynamics may therefore provide novel ways to treat inflammatory disease (Paszek et al. 2010).

NF-$\upkappa$B dynamics are generated via a complex network involving several negative feedback genes, such as A20 and I$\upkappa$B$\upalpha$ (Hoffmann and Baltimore 2006). Many mathematical models have been developed to recapitulate existing experimental data by quite complex biochemical networks involving up to 30 dynamic variables and 100 parameters with varying degrees of accuracy (Hoffmann et al. 2002; Lipniacki et al. 2004; Radulescu et al. 2008). Sensitivity analyses have then demonstrated that several parameters related to feedback regulation and IKK activation are responsible for generation of the oscillatory dynamics (Ihekwaba et al. 2004, 2005; Sung et al. 2009). An interesting extension of the sensitivity analysis method was proposed by Jacobsen and Cedersund (2008) who considered sensitivity with respect not just parameter perturbations but to variations of the network structure, e.g. introduction of delays in the network connections. Model reduction discussed in our paper provides an alternative avenue to extract core network components. Indeed, minimal models by Krishna *et al.* and Radulescu *et al.* demonstrated that part of this complex system in response to continuous cytokine stimulation may be reduced to three dynamical variables describing the nuclear NF-$\upkappa$B  and I$\upkappa$B$\upalpha$ mRNA and protein (Krishna et al. 2006). Here, we apply our method of speed coefficients to systematically reduce a 2-feedback model of the NF-$\upkappa$B system by Ashall et al. (2009).

Starting from a 14-variable model, we succeeded in closely representing dynamics of the NF-$\upkappa$B network in response to constant TNF$\upalpha$ input by a set of four variables (14). The minimal model included the nuclear NF-$\upkappa$B and its cytoplasmic inhibitor I$\upkappa$B$\alpha$, as well as two negative feedback loops represented by I$\upkappa$B$\upalpha$ and A20 transcripts. The latter variables were consistently ranked the slowest during successive reduction steps (Figs. 2, 5), and in fact their subsequent QSSA resulted in the loss of oscillations. This suggested that the timescale of transcription relative to other processes generates the key delayed negative feedback motif that drives oscillations in the system (Novak and Tyson 2008). While reducing the model, we observed that the period as well as the amplitude of oscillations was decreased with each reduction (Table 2). Replacing those variables with the respective QSSAs decreased the effective delay time in the system, and thus reduced the system’s propensity for oscillations. This effect was reverted by using first-order QSSA for some of the eliminated variables, namely cytoplasmic NF-$\upkappa$B, nuclear I$\upkappa$B$\upalpha$ and the active form of IKK kinase. A more accurate representation of those variables is thus important to faithfully represent NF-$\upkappa$B dynamics (Figs. 6, 7, 8). Our analysis is in agreement with results of Radulescu *et al.* who, using quasi-stationarity arguments, obtained a series of reduced models and eventually arrived at the 5 variable minimal model. While starting from a different two-feedback I$\upkappa$Ba and A20 model (Lipniacki et al. 2004) than the one considered here, Radulescu *et al.* showed similar requirements for both feedbacks to maintain oscillatory dynamics.

A model derived with respect to a specific solution is not necessarily able to reproduce the same breadth of responses as its forebear. However, by applying the algorithm with respect to a different solution one might try to potentially extract other key features of the system. Here, we demonstrated that the reduction of the model with respect to a pulsed and continuous TNF$\upalpha$ input resulted in a different order of elimination of the variables and ultimately a different minimal models (Fig. 9). The differences unravelled specific components of the IKK module responsible for NF-$\upkappa$B dynamics in response to different stimulus. With a pulsed input the amplitude of the subsequent peaks is determined by the “refractory period”, i.e. the time it takes for the active IKK to return to its neutral state. This requires a very accurate temporal representation of the neutral form of IKK, $v$, in the model. However, in response to continuous TNF$\upalpha$ input, both IKK-related variables became less important, and their steady-state approximation is sufficient to support the limit cycle. This analysis therefore begins to unravel how components of the KK signalling module could differentially encode temporal inflammatory signals.

In order to demonstrate a more general applicability of our method, we have employed the speed coefficient algorithm to derive a new reduced model of the Krishna model (Krishna et al. 2006). The comparison with minimal Krishna et al model showed that both models perform similarly in terms of time courses and phase portraits (Fig. 11). However, analysis of bifurcation diagrams showed that our algorithmic approach better preserved dynamical properties of the system (Fig. 12). In fact, the Krishna minimal model demonstrates features such as unstable limit cycle and hysteresis that are not present in the corresponding full model. Recently, Kourdis *et al.* used CSP algorithm to asymptotically analyse the dynamics of the Krishna *et al.* model. In agreement with our approach, their analysis identified similar fast/slow time scale variables that are essential to recapitulate limit cycle behaviour of the system. This analysis, in addition to our discussion of the Simplified Model, certainly suggests that our method has further potential as a viable technique for the reduction of biochemical network dynamic models.

Our objective here was to present and implement a new model reduction technique that without relying on prior biological insights, would preserve characteristics of the original model’s numerical solutions. This method thus belongs to a class of reduction methods that are algorithmic rather than biologically or biochemically intuitive, and as such should be applicable to complex biochemical models where the most important network sub-structures underlying the observed dynamical behaviour are not necessarily apparent. Similarly to other approximation methods, there is a trade-off between simplicity and accuracy of the end-point models. Even if errors introduced by one reduction step are small, for many steps they can accumulate. The approximations can be improved by using higher-order asymptotics, which increases algebraic complexity of the resulting reduced model but retains the dimensionality. We believe that in practically interesting cases, the increased algebraic complexity can be overcome by appropriate approximation of the functions in the resulting models. Another way to improve the accuracy of reduced models is to adjust parameters to match the solutions of the full model; a semi-empirical model resulting from such adjustment would still have an advantage over a fully empirical model in that at least its structure is not arbitrarily postulated. In addition to a lower dimensionality, the reduced problems are less stiff, as by definition, the variables with fastest characteristic timescales are eliminated first. The reduced dimensionality and stiffness allow, in principle, more efficient computations which may be important, e.g., for large scale models including interaction of many cells. Last but not least, systems of lower dimensionality are more amenable for qualitative study and intuitive understanding.

## Figures and Tables

**Fig. 13:**
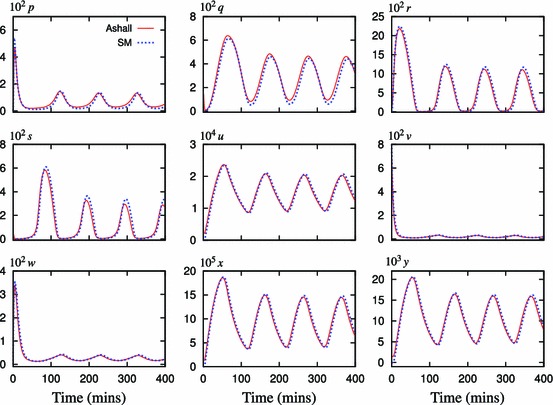
Ashall vs. Simplified Model. Time courses of solutions to the full Ashall model and the Simplified Model, in response to continuous TNF$\alpha$ treatment for time $t\ge 0$ ($t$ in minutes). Clearly the Simplified Model gives close agreement with the full model, in terms of variable amplitudes and the period of the limit cycle

## Appendix A: Simplified model vs. Ashall model

In Fig. 13, we show time courses of solutions to the full Ashall model and the Simplified Model, in response to continuous TNF$\alpha$ treatment, demonstrating the close agreement between the two models.

## Appendix B: Equations for the $z_0p_1y_1v_1s_1w_1$ model

The model consists of four ordinary differential equations

16a $$\begin{aligned} \frac{\mathrm {d}{q}}{\mathrm {d}{t}}&= -k_4q\bar{p}(q,r,x) +k_6u-k_8q-k_{13}q+k_{14}\bar{s}(q,r) -k_{17}\bar{w}(r,x)q,\end{aligned}$$

16b $$\begin{aligned} \frac{\mathrm {d}{r}}{\mathrm {d}{t}}&= k_{15}k_1\bar{p}(q,r,x) -k_4\bar{s}(q,r)r-k_{16}k_1r,\end{aligned}$$

16c $$\begin{aligned} \frac{\mathrm {d}{u}}{\mathrm {d}{t}}&= k_5\frac{r^{h}}{r^{h}+k^{h}} -k_7u,\end{aligned}$$

16d $$\begin{aligned} \frac{\mathrm {d}{x}}{\mathrm {d}{t}}&= k_9\frac{r^{h}}{r^{h}+k^{h}} -k_{11}x, \end{aligned}$$

where the functions in the right-hand side are defined by

17a $$\begin{aligned} w^{0}(x)&= \frac{ k_{24}k_{22}k_{20}k_{21}k_{12}k_3}{ \left( k_{20}k_{21}k_{12}+k_{24}k_{22}k_{21}k_{12}+k_{24}^{2}k_{22}k_{10}x\right) k_{23}}, \end{aligned}$$

17b $$\begin{aligned} w^{1}(r,x)&= \frac{ k_{24}^{3}k_{22}^{2}k_{20}k_{21}k_{12}k_3k_{10}\left( \frac{k_9r^{h}}{r^{h}+k^{h}}-k_{11}x\right) }{ k_{23}^{2}\left( k_{20}k_{21}k_{12}+k_{24}k_{22}k_{21}k_{12}+k_{24}^{2}k_{22}k_{10}x\right) ^{2} }, \end{aligned}$$

17c $$\begin{aligned} \bar{w}(r,x)&= w^{0}(x)+w^{1}(r,x) ,\end{aligned}$$

17d $$\begin{aligned} \bar{y}(r,x)&= \frac{k_{10}x}{k_{12}}-\frac{k_{12}(k_9\frac{r^{h}}{r^{h}+k^{h}}-k_{11}x)}{k_{10}^{2}},\end{aligned}$$

17e $$\begin{aligned} v^{0}(r,x)&= \frac{ k_3k_{20}k_{21}}{ k_{20}k_{21}+k_{21}k_{22}k_{24}+k_{24}^{2}k_{22}\bar{y}(r,x) },\end{aligned}$$

17f $$\begin{aligned} v^{1}(r,x)&= \frac{ -k_{20}^{2}k_{24}k_{10}k_3k_{21}^{2}(k_9\frac{r^{h}}{r^{h}+k^{h}}-k_{11}x) }{k_{12}(\frac{k_{20}k_{21}}{k_{21}+k_{24}\bar{y}(r,x)}+k_{24}k_{22})(\frac{k_{20}k_{21}}{k_{21}+k_{24}\bar{y}(r,x)}-k_{24}k_{22})^{2}(k_{21}+k_{24}\bar{y}(r,x))^{3} }, \nonumber \\ \end{aligned}$$

17g $$\begin{aligned} \bar{v}(r,x)&= v^{0}(r,x)+v^{1}(r,x),\end{aligned}$$

17h $$\begin{aligned} s_{\alpha }(q)&= k_1k_{13}q,\end{aligned}$$

17i $$\begin{aligned} s_{\beta }(r)&= k_4r+k_1k_{14}+k_{12},\end{aligned}$$

17j $$\begin{aligned} p^{0}(q,r,x)&= \frac{ k_{16}r+k_{18}\bar{w}(r,x)\left( k_3-\frac{r}{k_1}\right) }{ k_4q+k_{15}+k_{18}\bar{w}(r,x),}\end{aligned}$$

17k $$\begin{aligned} s_{\delta }(q,r,x)&= k_1k_{15}p^{0}(q,r,x)-k_1k_{16}r,\end{aligned}$$

17l $$\begin{aligned} s_{\gamma }(q,r,u,x)&= -k_4qp^{0}(q,r,x)-k_{13}q-k_8q+k_6u-k_{17}\bar{w}(r,x)q, \end{aligned}$$

17m $$\begin{aligned} \bar{s}(q,r,u,x)&= \frac{ s_{\alpha }(q)s_{\beta }(r)^{2}+k_4s_{\delta }(q,r,x)s_{\alpha }(q)-k_1k_{13}s_{\gamma }(q,r,u,x)s_{\beta }(r) }{ k_4^{2}s_{\alpha }(q)r+s_{\beta }^{3}(r)+k_1k_{13}k_{14}s_{\beta }(r)},\end{aligned}$$

17n $$\begin{aligned} \alpha _{p}(r,x)&= k_1k_{16}r+k_{18}\bar{w}(r,x)\left( k_1k_3-r\right) ,\end{aligned}$$

17o $$\begin{aligned} \beta _{p}(q,r,x)&= k_1\left( k_4q+ k_{15}+ k_{18}\bar{w}(r,x)\right) ,\end{aligned}$$

17p $$\begin{aligned} q_{\alpha }(q,r,u,x)&= k_6u- k_{13}q+ k_{14}s- k_8q- k_{17}\bar{w}(r,x)q,\end{aligned}$$

17q $$\begin{aligned} q_{\beta }(q)&= -k_4q,\end{aligned}$$

17r $$\begin{aligned} r_{\alpha }(q,r,u,x)&= -r\left( k_4\bar{s}(q,r,u,x) + k_{16}k_1\right) ,\end{aligned}$$

17s $$\begin{aligned} r_{\beta }&= k_1k_{15},\end{aligned}$$

17t $$\begin{aligned} p_{\delta }(r,x)&= k_1\left( k_4q_{\alpha }(q,r,u,x) + k_{18}\left( k_{24}k_{22}\bar{v}(r,x) - k_{23}\bar{w}(r,x)\right) \right) ,\end{aligned}$$

17u $$\begin{aligned} p_{\gamma }(q)&= k_1k_4q_{\beta }(q,r,u,x),\end{aligned}$$

17v $$\begin{aligned} p_{\varepsilon }(q,r,u,x)&= k_{16}k_1r_{\alpha }(q,r,u,x)-k_{18}\bar{w}(r,x) r_{\alpha }(q,r,u,x), \end{aligned}$$

17w $$\begin{aligned}&\quad +k_{18}\left( k_{24}k_{22}\bar{v}(r,x)-k_{23}\bar{w}(r,x)\right) \left( k_1k_3- r\right) ,\end{aligned}$$

17x $$\begin{aligned} p_{\zeta }(r,x)&= \left( k_1k_{16}-k_{18}\bar{w}(r,x)\right) r_{\beta },\end{aligned}$$

17y $$\begin{aligned} \bar{p}(q,r,u,x)&= \frac{\beta _{p}^{2}\alpha _{p}+\alpha _{p}p_{\delta }-\beta _{p}p_{\varepsilon }}{\beta _{p}^{3}-\alpha _{p}p_{\gamma }+\beta _{p}p_{\zeta }}. \end{aligned}$$

## Appendix C: Equations for the $z_0p_0y_0v_0$ model

Dynamic equations for the 6-variable model, $z_0p_0y_0v_0$, reduced using a representative solution for continuous TNF$\upalpha$ stimulation ($k_{24}\equiv 1$):

18a $$\begin{aligned} \frac{\mathrm {d}{q}}{\mathrm {d}{t}}&= -k_4q\bar{p}+k_6u-k_8q-k_{13}q+k_{14}s-k_{17}\bar{w}q\end{aligned}$$

18b $$\begin{aligned} \frac{\mathrm {d}{r}}{\mathrm {d}{t}}&= k_{15}k_1\bar{p}-k_4sr-k_{16}k_1r\end{aligned}$$

18c $$\begin{aligned} \frac{\mathrm {d}{s}}{\mathrm {d}{t}}&= k_{13}k_1\bar{p}-k_4sr-k_8s-k_{14}k_1s\end{aligned}$$

18d $$\begin{aligned} \frac{\mathrm {d}{u}}{\mathrm {d}{t}}&= k_5\frac{r^{h}}{r^{h}+k^{h}}-k_7u\end{aligned}$$

18e $$\begin{aligned} \frac{\mathrm {d}{w}}{\mathrm {d}{t}}&= k_{24}k_{22}v-k_{23}w\end{aligned}$$

18f $$\begin{aligned} \frac{\mathrm {d}{x}}{\mathrm {d}{t}}&= k_9\frac{r^{h}}{r^{h}+k^{h}}-k_{11}x\end{aligned}$$

18g $$\begin{aligned} \bar{p}&= \frac{k_{16}rk_1+k_{18}\bar{w}k_3k_1-k_{18}\bar{w}r}{k_1\left( k_4q+k_{15}+k_{18}\bar{w}\right) }\end{aligned}$$

18h $$\begin{aligned} \bar{y}&= \frac{k_{10}x}{k_{12}}\end{aligned}$$

18i $$\begin{aligned} \bar{v}&= \frac{k_2k_{20}k_{21}}{k_{21}k_{22}k_{24}+k_{24}^2k_{22}y+k_{20}k_{21}} \end{aligned}$$

## Appendix D: Equations for K6, K3 and K4 models

### D.1 Krishna full model (K6)

The full Krishna (Krishna et al. 2006) 7-variable model for $N_{n}$ and $N$ (free nuclear and cytoplasmic NF-$\upkappa$B  $I_{m}$ (I$\upkappa$B$\upalpha$ mRNA), $I_{n}$ and $I$ (free nuclear and cytoplasmic I$\upkappa$B), $(NI)_{n}$ and $(NI)$ (nuclear and cytoplasmic NF-$\upkappa$B:I$\upkappa$B$\upalpha$) is given as:

19a $$\begin{aligned} \frac{\mathrm {d}{N_{n}}}{\mathrm {d}{t}}&= k_{Nin}N - k_{fn}N_{n}I_{n} + k_{bn}(NI)_{n}, \end{aligned}$$

19b $$\begin{aligned} \frac{\mathrm {d}{I_{m}}}{\mathrm {d}{t}}&= k_{t}N_{n}^{2} - \gamma _{m}I_{m}, \end{aligned}$$

19c $$\begin{aligned} \frac{\mathrm {d}{I}}{\mathrm {d}{t}}&= k_{tl}I_{m} - k_{f}N I + k_{b}(NI) - k_{Iin} I + k_{Iout}I_{n}, \end{aligned}$$

19d $$\begin{aligned} \frac{\mathrm {d}{N}}{\mathrm {d}{t}}&= -k_{f}N I + (k_{b}+\alpha )(NI) - k_{Nin}N, \end{aligned}$$

19e $$\begin{aligned} \frac{\mathrm {d}{(NI)}}{\mathrm {d}{t}}&= k_{f}N I - (k_{b}+\alpha )(NI) + k_{NIout}(NI)_{n}, \end{aligned}$$

19f $$\begin{aligned} \frac{\mathrm {d}{I_{n}}}{\mathrm {d}{t}}&= k_{Iin}I - k_{Iout}I_{n} - k_{fn}N_{n}I_{n} + k_{bn}(NI)_{n}, \end{aligned}$$

19g $$\begin{aligned} \frac{\mathrm {d}{(NI)_{n}}}{\mathrm {d}{t}}&= k_{fn}N_{n}I_{n} - (k_{bn}+k_{NIout})(NI)_{n}. \end{aligned}$$

Note that this can be replaced by a 6-variable system by using conservation of NF-$\upkappa$B to eliminate $N$. Base parameter values used in Krishna et al. (2006) are given in Table 3.

**Table 3:** Parameter values for Krishna model

Parameter	Value	Units	Parameter	Value	Units
$k_{Nin}$	5.4	min$^{-1}$	$k_{fn}$	30	$\upmu \text {M}^{-1}\,\text {min}^{-1}$
$k_{bn}$	0.03	min$^{-1}$	$k_{t}$	1.03	$\upmu \text {M}^{-1}\,\text {min}^{-1}$
$\gamma _{m}$	0.017	min$^{-1}$	$k_{tl}$	0.24	min$^{-1}$
$k_{f}$	30	$\upmu \text {M}^{-1}\,\text {min}^{-1}$	$k_{b}$	0.03	
$k_{Iin}$	0.018	min$^{-1}$	$k_{Iout}$	0.012	min$^{-1}$
$\alpha$	1.05 IKK	min$^{-1}$	$k_{NIout}$	0.83	min$^{-1}$
IKK	0.5	$\upmu \text {M}$	$N_{tot}$	1	$\upmu \text {M}$

### D.2 Krishna 3-variable model (K3)

The Krishna (Krishna et al. 2006) reduced model has three variables, and is given (in their Supplementary Material) as follows:

20a $$\begin{aligned} \frac{\mathrm {d}{N_{n}}}{\mathrm {d}{t}}&= k_{Nin}K_{I}\frac{N_{tot}-N_{n}}{K_{I}+I} - k_{Iin} \frac{I N_{n}}{\delta + N_{n}}, \end{aligned}$$

20b $$\begin{aligned} \frac{\mathrm {d}{I_{m}}}{\mathrm {d}{t}}&= k_{t}N_{n}^{2} - \gamma _{m} I_{m}, \end{aligned}$$

20c $$\begin{aligned} \frac{\mathrm {d}{I}}{\mathrm {d}{t}}&= k_{tl}I_{m} - \alpha \frac{N_{tot}-N_{n}}{K_{I}+I} I, \end{aligned}$$

where

21 $$\begin{aligned} K_{I}=\frac{k_{b}+\alpha }{k_{f}}, \qquad \qquad K_{N}=\frac{k_{bn}+K_{NIout}}{k_{fn}}, \qquad \qquad \delta =\frac{K_{N}}{N_{tot}}. \end{aligned}$$

### D.3 New 4-variable model

The 4-variable model, obtained by applying our speed coefficient algorithm to K6 (with $N$ eliminated), comes from first-order QSSA for $N_{n}$ followed by zeroth-order QSSA for $(NI)_{n}$. The resulting system for variables $I_{m},\;I,\;(NI)$ and $I_{n}$ is given by:

22a $$\begin{aligned} \frac{\mathrm {d}{I_{m}}}{\mathrm {d}{t}}&= k_{t}N_{n}^{2} \qquad - \gamma _{m}I_{m},\end{aligned}$$

22b $$\begin{aligned} \frac{\mathrm {d}{I}}{\mathrm {d}{t}}&= \left[ k_{tl}I_{m} + k_{b}(NI) + k_{Iout}I_{n}\right] - \left\{ k_{f}\left[ N_{tot}-(NI)-N_{n}-(NI)_{n}\right] + k_{Iin} \right\} I, \nonumber \\ \end{aligned}$$

22c $$\begin{aligned} \frac{\mathrm {d}{(NI)}}{\mathrm {d}{t}}&= \left[ k_{f}I \left\{ N_{tot}-N_{n}-(NI)_{n}\right\} + k_{NIout}(NI)_{n} \right] \qquad - (k_{f}I+k_{b}+\alpha ) (NI),\nonumber \\ \end{aligned}$$

22d $$\begin{aligned} \frac{\mathrm {d}{I_{n}}}{\mathrm {d}{t}}&= \left[ k_{Iin}I + k_{bn}(NI)_{n}\right] \qquad - (k_{Iout}+k_{fn}N_{n}) I_{n}, \end{aligned}$$

with

22e $$\begin{aligned} N_{n}&= \frac{-b-\sqrt{b^{2}-4ac}}{2a}, \end{aligned}$$

22f $$\begin{aligned} (NI)_{n}&= \frac{k_{fn}N_{n}I_{n}}{k_{bn}+k_{NIout}}, \end{aligned}$$

where

22g $$\begin{aligned} N_{tot}&= N + N_{n} + (NI)_{n} + (NI), \end{aligned}$$

22h $$\begin{aligned} a&= k_{NIout}k_{fn}^{3}(k_{Nin}-k_{bn}) I_{n}^{2}, \end{aligned}$$

22i $$\begin{aligned} b&= -\Big (k_{bn} + k_{NIout}\Big ) \;\;\; \Big \{ I_{n} k_{NIout} k_{Nin} k_{fn}^{2} + k_{Nin}^{3} k_{NIout} + 2 k_{Nin} k_{NIout} k_{fn}^{2} I_{n}^{2} \nonumber \\&\quad +\,\, k_{NIout} k_{Nin}^{2} k_{f} I + k_{NIout} k_{Nin} k_{fn} I_{n} k_{f}I + k_{NIout} k_{fn}^{3} I_{n}^{3} \nonumber \\&\quad +\,\, 2 k_{Nin}^{2} k_{NIout} k_{fn} I_{n} - k_{NIout} k_{Nin} (NI) I_{n} k_{fn}^{2} + 2 k_{Nin}^{2} k_{bn} k_{fn} I_{n} \nonumber \\&\quad +\,\, k_{Nin} k_{bn} k_{fn}^{2} I_{n}^{2} \nonumber \\&\quad +\,\, k_{Nin}^{2} k_{f} I k_{bn} - k_{Nin} k_{fn}^{2} I_{n}^{2} k_{IouI_{n}} + k_{bn} k_{fn}^{2} I_{n}^{2} k_{IouI_{n}} + k_{Nin}^{3} k_{bn} \nonumber \\&\quad +\,\, k_{Nin} k_{fn} I_{n} k_{f} I k_{bn} \nonumber \\&\quad +\,\, k_{Nin}^{3} k_{fn} I_{n} + k_{Nin} k_{fn}^{3} I_{n}^{3} + 2 k_{Nin}^{2} k_{fn}^{2} I_{n}^{2} + k_{Nin} k_{fn}^{2} I_{n} k_{Iin} I - k_{bn} k_{fn}^{2} I_{n} k_{Iin} I \nonumber \\&\quad +\,\, k_{Nin}^{2} k_{f} I k_{fn} I_{n} + k_{Nin} k_{fn} ^2 I_{n}^{2} k_{f} I \Big \}, \end{aligned}$$

22j $$\begin{aligned} c&= -k_{Nin} \Big (k_{bn} + k_{NIout}\Big )^{2} \;\;\; \Big \{ -k_{Nin}^{2} + k_{Nin}^{2} (NI) - 2 k_{Nin} k_{fn} I_{n} + k_{Nin} (NI) k_{b} \nonumber \\&\quad + k_{Nin} (NI) \alpha + 2 k_{Nin} (NI) k_{fn} I_{n} - k_{Nin} k_{f} I + k_{Nin} (NI) k_{f} I \nonumber \\&\quad +\,\, k_{fn} I_{n} kIo - k_{fn} (NI) I_{n} kIo + k_{fn} I_{n} (NI) k_{f} I + k_{fn} (NI) k_{Iin} I - k_{f} I k_{fn} I_{n} \nonumber \\&\quad +\,\, k_{fn} I_{n} (NI) k_{b} - k_{fn}^{2} I_{n} ^{2} + k_{fn} I_{n} (NI) \alpha + (NI) k_{fn}^{2} I_{n}^{2} - k_{fn} k_{Iin} I \Big \}. \end{aligned}$$
